# Gyroscope-Driven Mouse Pointer with an EMOTIV^®^ EEG Headset and Data Analysis Based on Empirical Mode Decomposition

**DOI:** 10.3390/s130810561

**Published:** 2013-08-14

**Authors:** Gerardo Rosas-Cholula, Juan Manuel Ramirez-Cortes, Vicente Alarcon-Aquino, Pilar Gomez-Gil, Jose de Jesus Rangel-Magdaleno, Carlos Reyes-Garcia

**Affiliations:** 1 Department of Electronics, National Institute of Astrophysics, Optics and Electronics, Luis Enrique Erro No. 1, Tonantzintla, Puebla 72760, Mexico; E-Mails: grosas@inaoep.mx (G.R.-C.); jrangel@inaoep.mx (J.J.R.-M.); 2 Department of Electronics and Computer Science, Exhda. Sta. Catarina Martir, Cholula, University of the Americas, Puebla, Puebla 72720, Mexico; E-Mail: vicente.alarcon@udlap.mx; 3 Department of Computer Science, National Institute of Astrophysics, Optics and Electronics, Luis Enrique Erro No. 1, Tonantzintla, Puebla 72760, Mexico; E-Mails: pgomez@inaoep.mx (P.G.-G.); kargaxxi@inaoep.mx (C.R.-G.)

**Keywords:** gyroscope-driven, empirical mode decomposition, Kalman filter, electroencephalographic signals

## Abstract

This paper presents a project on the development of a cursor control emulating the typical operations of a computer-mouse, using gyroscope and eye-blinking electromyographic signals which are obtained through a commercial 16-electrode wireless headset, recently released by Emotiv. The cursor position is controlled using information from a gyroscope included in the headset. The clicks are generated through the user's blinking with an adequate detection procedure based on the spectral-like technique called Empirical Mode Decomposition (EMD). EMD is proposed as a simple and quick computational tool, yet effective, aimed to artifact reduction from head movements as well as a method to detect blinking signals for mouse control. Kalman filter is used as state estimator for mouse position control and jitter removal. The detection rate obtained in average was 94.9%. Experimental setup and some obtained results are presented.

## 1. Introduction

In the last decades there has been a growing effort from the research community to develop human-computer interfaces (HCI), aiming to provide convenient communication alternatives for disabled persons. Several approaches of user-friendly interfaces using voice, vision, gesture, and other modalities, can be found in recent literature [1–6]. As the technology advances, new affordable devices make attractive the use of bioelectric signals such as electroencephalographic (EEG), electromyographic (EMG), or electro-oculographic signals (EOG), for the purpose of developing new types of HCI systems [7–10]. Among these, EEG have received considerable attention due to several factors arising on practical scenarios, such as noninvasiveness, portability, and relative cost, without lost on accuracy and generalization. An important motivation to develop user-friendly HCI systems, among some others, is to allow an individual with severe motor disabilities to have control over specialized devices such as assistive appliances, neural prostheses, speech synthesizers, or a personal computer directly. The standard computer interface nowadays involves a keyboard and mouse, although recently, touchscreen interfaces are becoming very popular. In that sense, computer mouse emulation using hands-free alternatives is still a very helpful resource for disabled persons. The following is a partial list of some successful modalities which have been reported: visual tracking [5], voice control [6], electromyographic signals [7], electro-oculographic potentials (EOG) [8], and electroencephalographic signals [9,10].

In this work we present a project focusing on the development of a hands-free mouse emulation using the EEG headset recently released by Emotiv [11]. The Emotiv EPOC headset represents a practical, economical, and efficient alternative for EEG-based applications, which has been recently used in a number of applications such as emotions detection supporting instant messaging [12], visual imagery for classification of primitive shapes [13], P300 rhythm detection [14], or a human-machine interface applied into a tractor steering [15]. In the project described in this paper, the Emotiv headset is used to emulate the basic operations of a mouse computer, with position control based on the speed signals obtained from a gyroscope included in the headset. The clicks are carried out through the user's blinking, which generates electrical activity originated in muscle movements in the form of EMG signals, and detected through the headset electrodes. For that purpose, Empirical Mode Decomposition (EMD) is explored as a separation technique in order to locate in time and pseudo-spectrum, the energy of the associated pulses from a background with noise and other artifacts. There are several successful approaches reported in the literature aiming to separation of blinking signals from EEG measurements, using techniques such as Independent Component Analysis (ICA) [16,17], wavelet analysis [18,19], a combination of the two previous [20], algebraic separation [21], and Hilbert-Huang transform (HHT) [22]. Most references found in the literature refer to blinking signals separation in the context of artifact removal, although there are some studies on the use of blinking signals for control applications [23–25]. Reference [25] presents a machine learning approach to detect eye movements and blinks to control an external device, using Common Spatial Pattern (CSP) filters during feature extraction, with an accuracy of 95%. Reference [26] presents a comparison of Discrete Wavelet Transform (DWT) and Hilbert-Huang Transform (HHT), both used in EEG signal feature extraction. HHT was reported to provide a more accurate time-frequency analysis due to its adaptive basis according to the input data. Additionally, when DWT is used, the choice of a suitable wavelet and threshold values is a crucial task to be considered [27]. Independent Component Analysis (ICA) has been widely used in EEG analysis for artifacts removal, SNR enhancement, and optimal electrodes selection [28,29], however, some minor drawbacks such as power spectrum corruption [30], or component localization [31] may be present. According to the references consulted, ICA has been mainly used in offline analysis. Detection and removal of myogenic and ocular artifacts using ICA is extensively analyzed in [31]. A relevant discussion presented in this reference is how to objectively identify components related to ocular artifacts, as this is often done based on visual inspection, thus relying on the subjective judgment of the experimenter. Blind source separation delivers separated independent components in no specific order, making difficult implementation of real time automatic systems. Additional information is included in some studies in order to increase ICA performance, such as eye tracking signals [31] or video sequences [32]. Although there are several developed approaches, direct performance comparisons between reported methods is not straightforward given that in many cases EOG blinking signals used for controlling some devices, are measured through electrodes located around the ocular globe, where power signal is higher and in consequence easier to detect using techniques as simple thresholding. The experimental prototype described in this paper has been designed specifically with the goal of using the functionalities provided by the EMOTIV wireless EEG headset. Although Emotiv EPOC headset represents a practical alternative for the development of accessible EEG-based applications, the obtained signals present usually poor signal to noise ratio, and contacts between electrodes and scalp generate noise arising even from head movements. The application reported in this work is based on the use of EMD as an adequate technique aiming to signal detection of EMG blinking signals through the mentioned Emotiv EPOC headset. Table 1 summarizes some techniques of preprocessing, feature extraction, and classification, recently reported applied to blinking detection using electrodes as element sensor.

EMD is a technique used to decompose a time series into a finite number of functions called intrinsic mode functions (IMF) using an empirical identification based on its characteristic time scales [51]. EMD has been recently proposed as an analysis tool in a number of applications such as, image texture analysis [52], detection of human cataract in ultrasound signals [53], crackle sounds analysis [54], vibration signal analysis for localized gearbox fault diagnosis [55], image watermarking [56], and EEG signal analysis. Examples of the last category, closely related to the topic of the presented paper are event related potentials (ERP) [57], phase synchrony measurement from the complex motor imaginary potential of combined body and limb action [58], and EEG signals for synchronization analysis [59]. From the point of view of the EOG, the eye can be modeled as a fixed dipole with positive pole at the cornea and negative pole at the retina. The potential of this dipole is known as corneo-retinal potential, with typical amplitude values in the range of 0.4–1.0 mV. Eye movements produce rotating dipole and consequently potential signals proportional to the movement appear. These signals can be relatively easy to acquire. There are several methods for determining eye movement such as infrared oculography, which uses corneal reflection of near infrared light with the pupil center as reference location [60], video oculography [61], magnetic field search coil technique [62], and others. EOG is an effective and direct method to detect eye movements. The main disadvantage applying EOG is that the corneoretinal potential is not always fixed and depends on diverse factors, for instance, diurnal variations, fatigue, and intensity of light. In consequence frequent calibration is needed.

In this work, a feature extraction procedure based on the spectral-like technique EMD is described. Results obtained using the proposed technique indicate an adequate process to hand the non-stationarity characteristic of EEG signals. Additional experiments using DWT were carried out for comparison purposes. Typical mouse-like function is a sequential process in which the user performs a movement to locate the cursor in the required position, and then selects an operation by applying a click action. Experiments were carried out considering extreme situations, where the subject is instructed to move the head at different speeds while applying a double click in indicated times. Section 2 describes some theoretical background on the used techniques. Section 3 presents a description of the experimental setup. Section 4 describes some obtained results, and section 5 presents some concluding remarks and future work about the described project.

## 2. Empirical Mode Decomposition (EMD)

EMD was first introduced by Huang [51] for spectral analysis of non-linear and non-stationary time series, as the first step of a two stage process, currently known as the Hilbert Huang Transform (HHT). EMD is used in this work with two objectives: signal preprocessing to reduce noise arising from head movement, and double blinking detection to simulate the “click” operation of a traditional mouse device. Essentially, EMD aims to empirically identify the intrinsic oscillatory modes or intrinsic mode functions (IMF) of a signal by its characteristic time scales, in adaptive way. These modes represent the data by means of local zero mean oscillating waves obtained by a sifting process. Thus, an IMF satisfies two main conditions: taking account the complete data set, the number of extrema points (min and max) must be equal or differ at most by one to the number of zero crossing points; the mean value of the envelopes is always zero which are defined by the local maxima and local minima. EMD can be summarized as follows (see [63] for details): Given a signal *x*(*t*) (*t* is the time) identify its extrema (both minima *e*_min(_*_t_*_)_ and maxima *e*_max(_*_t_*_)_). Generate the envelope by connecting maxima and minima points with a curve, for instance, cubic spline interpolation, although other interpolation techniques are allowed. Determine the mean by averaging; Equation (1). Extract the detail *d*; Equation (2). Finally iterate on the residual *m*(*t*):

(1) $$m(t)=\frac{e_{\min(t)}+e_{\max(t)}}{2}$$

(2) d(t)=x(t)−m(t)

There are iteration stopping criteria such as establishing a certain number of siftings, thresholds, or minimum amplitude of residual. EMD satisfies completeness and orthogonality properties in the same way as spectral decompositions such as Fourier or wavelet transform. The completeness property is satisfied by EMD, in the sense that it is possible to reconstruct the original signal based on their decompositions. These decomposition functions should all be locally orthogonal to each other, as expressed in Equation (3), although some leakage may arise:

(3) $$\bar{(x(t)-\bar{x(t)})•\bar{x(t)}}=0$$

An orthogonality index expressed in Equation (4) is used to keep track of leakage magnitude of some limits. *X* is the original signal with *i* ≠ *j*, where *n* is the number of decompositions and *T* is the number of samples inside the analysis window:

(4) $$I_{O}=\sum_{t=0}^{T}\left(\frac{\sum_{j=1}^{n+1}\sum_{k=1}^{n+1}IMF_{j}\;(t)IMF_{k}\;(t)}{X^{2}(t)}\right)$$

Occasionally, the consideration of a local EMD is necessary. In this case, sifting operations are not applied to the full length signal. Sometimes, a better local approximation is obtaining through over-iteration of a specific zone; however, this process produces contamination in other signal zones and in consequence over-decomposing. Thus, the algorithm must keep iterating only over zones where the error remains large. Local EMD is implemented introducing a weighting function (*w(t*)), that describes a soft decay outside the problem zone. In consequence Equation (2) can be written as:

(5) d(t)=x(t)−w(t)m(t)

**Figure 1.**
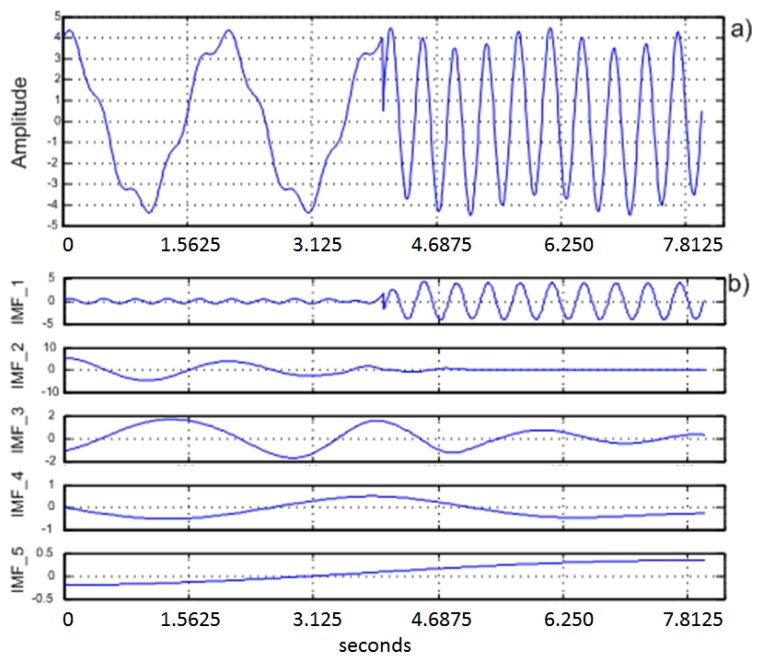
(**a**) Original EEG signal, (**b**) first five IMFs.

Figure 1 shows typical results obtained from an EEG signal using EMD with five decomposition iterations.

## 3. Proposed Scheme and Module Description

**Figure 2.**
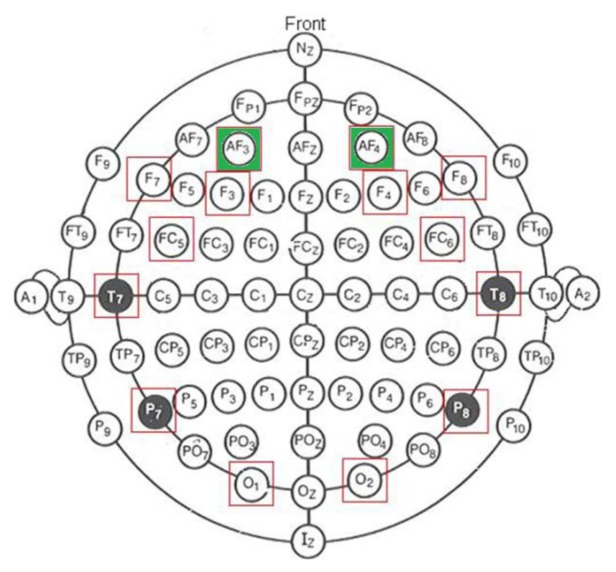
International system 10–20.

In EEG signal detection, it is important to get consistent records of electric brain activity from specific surface electrode location. For that purpose, scientists and physicians rely on a standard system for accurately placing electrodes, which is called the International 10–20 System, generally used in clinical EEG recording and EEG research. Figure 2 shows the electrode positions and denominations used in the International 10–20 System. Red squares indicate the available electrodes on Emotiv system. The EEG signals required to perform the detection are obtained from electrodes AF3/AF4 (green marked in Figure 2), which are labeled according to the mentioned 10–20 International System.

**Figure 3.**
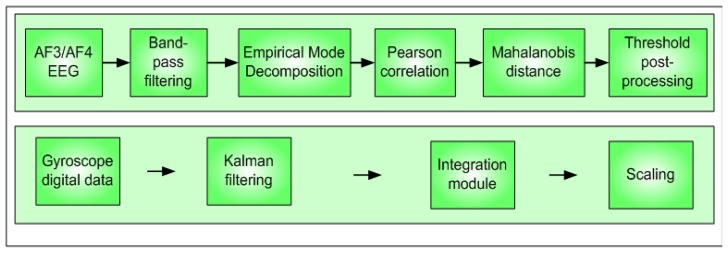
Proposed scheme, blinking detection and gyroscope processing system.

The modules proposed to detect double-blinking event and to process gyroscope data are shown in the block diagram of Figure 3. A preprocessing stage using a band-pass filter (0.5 Hz–10 Hz) is applied before doing the spectral analysis.

### 3.1. Noise Reduction

**Figure 4.**
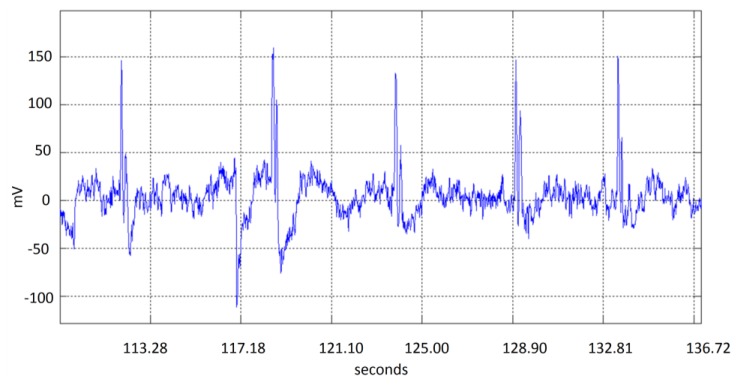
Head movement noise during double blinking events.

**Figure 5.**
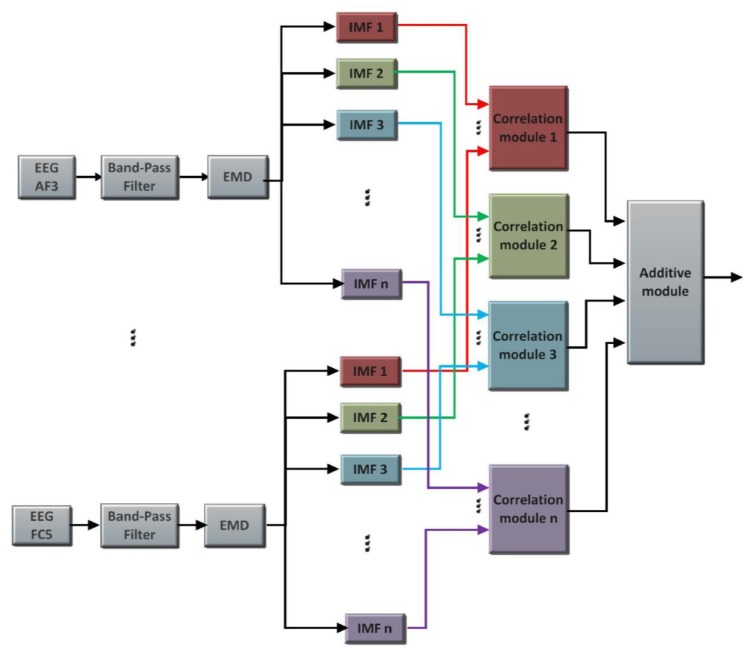
Preprocessing to reduce head movement noise.

EEG signals provided by the EMOTIV headset EEG acquisition system are contaminated by noise produced by different sources such as: muscular movements (head movement, breathing, *etc*.), or electromagnetic noise (50/60 Hz electric lines). Although Emotiv EPOC headset represents an efficient, practical and economical alternative, the EEG detected signals are often noisy. Head movements associated to the expected use of the device as a mouse pointer will produce noise on the signal acquired by the electrode due to a slight movement of the electrode over the scalp. Figure 4 shows five double blinking events immerse in noise produced by head movement, which can occur even in the same order of magnitude than expected blinking amplitude values. The artifact could be detected considering that noise present in all electrodes over the scalp will show high correlation. Thus a preprocessing stage includes finding common signals in the electrodes. The preprocessing consists of EMD decomposition, correlation based function and an integration module, as described in Figure 5.

As previously stated, noise produced by head or body movement will appear in all electrodes of the system with small variations, therefore, correlation analysis using Pearson coefficient is used for noise detection purposes. Pearson correlation coefficient provides a measure of dependence between two random variables. Equation (6) defines the Pearson correlation with expected values *μ_X_* and *μ_Y_* and standard deviations *σ_X_* and *σ_Y_*:

(6) $$\rho_{↓}(X,Y)=\frac{E\left[\left(X-\mu_{↓}X\right)\left(Y-\mu_{↓}Y\right)\right]}{\left(\sigma_{↓}X\sigma_{↓}Y\right)}$$

**Figure 6.**
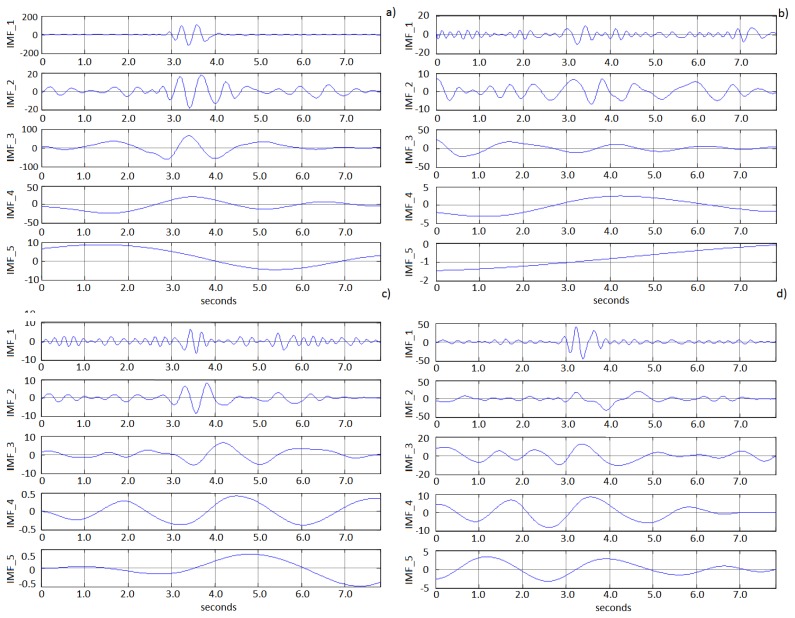
EMD decomposition from four different electrodes near AF3. (**a**) FC5, (**b**) FC6, (**c**) P8, and (**d**) P7.

Correlation function applied directly to the signals obtained from each electrode will state dependence between channels. Common signals detected would have to be removed; however, applying directly an operation to separate those signals could cause removing also important information. Therefore, decomposing the signal from each electrode will reduce the loss of information, allowing the system to distinguish between artifacts from head movements and double blinking signals. That decomposition has been carried out using EMD technique. Figure 6 shows an example of EMD decomposition, with a plot of IMF 1 to IMF 5 obtained from four different electrodes near AF3. Visual inspection indicates similarities in IMFs 1, 3, 4 and 5. In this part of the experiment, EMD decomposition typically yielded between 14 and 16 IMFs.

**Figure 7.**
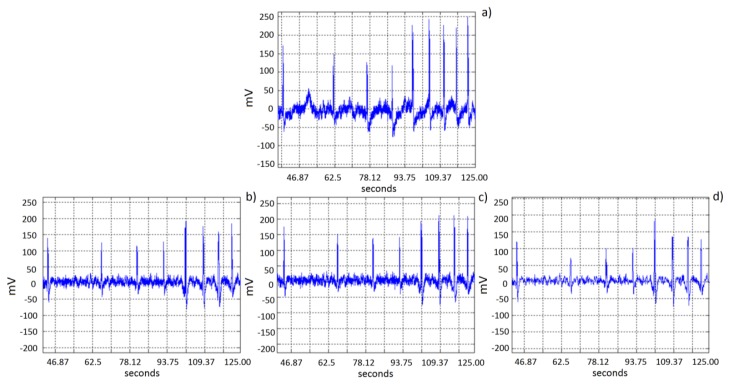
Noise reduction based on correlation function removing, (**a**) 1 IMF, (**b**) 2 IMFs, (**c**) 3 IMFs and (**d**) 4 IMFs.

In order to find the amount of similarity or dependence, the Pearson correlation is calculated from corresponding IMF functions. Additionally, a *p*-value is computed by transforming the correlation to create a t statistic with *n-2* degrees of freedom, where *n* is the number of rows in the correlation matrix. Thus, *p*-values less than 0.05 were considered to imply high correlation. Figure 6 shows an example in which IMF3 from electrode AF6 is compared to the rest, from a total number of 12 electrodes, resulting in *p*-values close to 0, except for FC6 electrode (0.639). This algorithm is repeated for all IMFs,taking as reference the electrode AF6. A slide window of 10 s is applied during correlation calculation. Figure 7 shows the noise reduction using the correlation coefficients associated to the corresponding IMF. If there is a correlation in most of the electrodes, the corresponding IMF is prevented from passing to the integration module.

**Figure 8.**
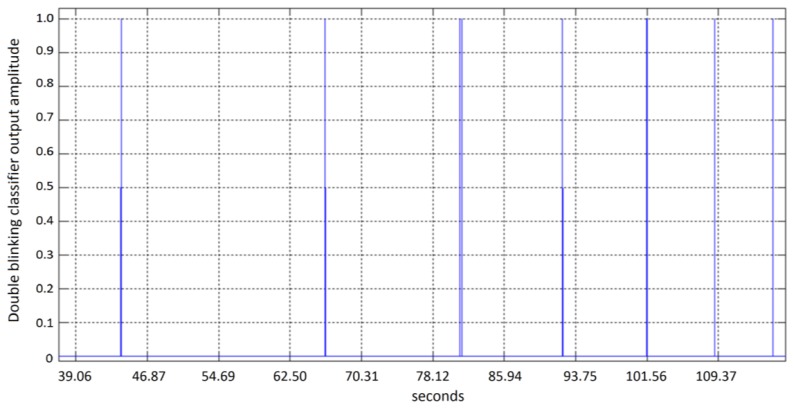
Double blinking detection with noise reduction.

Once the noise is reduced, a second derivative is obtained in order to determine whether a critical point is a local maximum or a local minimum. A typical double blinking event will have two local max points inside a 0.5 s window. Figure 8 shows the signal after this processing, thus the classifier is reduced to a simple threshold function.

### 3.2. Pointer Movement

**Figure 9.**
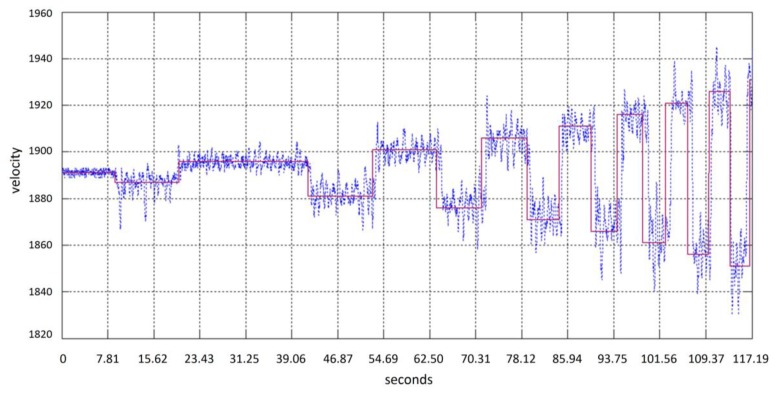
Gyroscope data and velocity target movement from subject head movement; target movement (red line), head movement (blue line).

**Figure 10.**
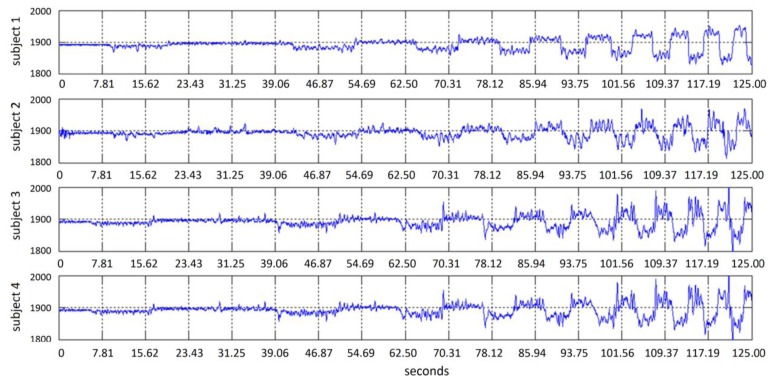
Gyroscope data and velocity target movement from four different subjects head movements.

The gyroscope IC embedded in the Emotiv headset provides information about head movements through a speed signal. An integration step is then required in order to obtain the cursor relative position. Figure 9 shows a typical gyroscope signal obtained when the subject moves the head, following the test point in the screen in an oscillating horizontal way with an increasing speed. Figure 10 shows signals obtained from 4 different subjects. A simple scaling stage is needed to adjust screen resolution and sensibility. Testing system covers linear velocity movements in the range of 0 to 455 pixels per second.

In this stage, Kalman filter was used as a state estimator for mouse position control and jitter removal. Kalman filter is a recursive estimator that is used for computing a future estimate of the dynamic system state from a series of noisy measurements, minimizing the mean of the squared estimate error, between the prediction of the system's state and the measurement. Estimated state from the previous time step and the new measurements are used to compute a new estimate for the current dynamic system's state. Kalman filter has been used as estimator to perform smooth tracking and jitter remotion in several contexts such as image stabilization [64], real time face-tracking [65], and robot vision [66]. Kalman matrixes (Equations (9) and (10)) are obtained considering a model similar to a particle moving in the plane at constant velocity subject to random perturbations in its trajectory, as expressed in Equations (7) and (8), in its discrete form, where *T_s_* is the sampling period:

(7) $$\left[\begin{array}{c} x_{1}\\ x_{2}\\ {\dot{x}}_{1}\\ {\dot{x}}_{2} \end{array}\right]=\left[\begin{array}{cccc} 1 & 0 & T_{s} & 0\\ 0 & 1 & 0 & T_{s}\\ 0 & 0 & 1 & 0\\ 0 & 0 & 0 & 1 \end{array}\right]\;x+w$$

(8) $$\left[\begin{array}{c} z_{1}\\ z_{2} \end{array}\right]=\left[\begin{array}{cccc} 1 & 0 & 0 & 0\\ 0 & 1 & 0 & 0 \end{array}\right]\;x+v$$

The Kalman filter is a recursive predictive filter that utilizes minimization of covariance error becoming it in an optimal estimator. The filter is based on the system definition using state space variables and recursive algorithms for the minimization process [67]. The filter consists of two steps: prediction and correction. The prediction or priori state solves the differential equations that describe the dynamic model, as represented in Equation (9), where ***x*** is the state vector, ***F*** the system dynamics matrix and ***w*** is a white noise process. The measurements are linearly related to the states according to Equation (10), which is known as the observation model, where ***z*** is the measurement vector, ***H*** is the measurement matrix and ***v*** is the white measurement noise vector. The solution of the differential equations is a linear combination of the initial state ***x*** which is described by Equation (11), where Φ is the fundamental matrix:

(9) x=Fx+w

(10) z=Hx+v

(11) $$x(t)=\Phi (t-t_{0})x(t_{0})$$

**Figure 11.**
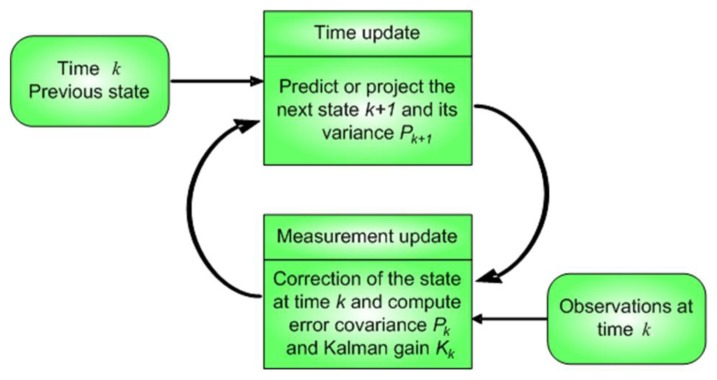
Simplified flow diagram for Kalman filter.

Figure 11 shows the recursive process followed by Kalman filter. Projection of the state vector at time *k* + *1* is improved using the observation at time *k* in such a way that the error covariance of the estimator is minimized. Kalman gain is obtained from the Ricatti equations given by Equations (12–14), which are a set of recursive matrix equations:

(12) $$M_{k}=\phi_{k}P_{k-1}\Phi_{k}^{T}+Q_{k}$$

(13) $$K_{k}=M_{k}H^{T}{\left(HM_{k}H^{T}+R_{k}\right)}^{-1}$$

(14) $$P_{k}=(I-K_{k}H)M_{k}\;0$$

where *P_k_* is a covariance matrix representing errors in the state estimates after an update, and *M_k_* is the covariance matrix representing errors in the state estimates before an update. *Q* represents the process noise matrix, which is related to the process-noise vector according to Equation (15). In the same manner, matrix *R* is related to the measurement noise vector *v* according to Equation (16), considering zero mean noise gaussian distribution [59]:

(15) $$Q=E\left[ww^{T}\right]$$

(16) $$R=E\left[vv^{T}\right]$$

**Figure 12.**
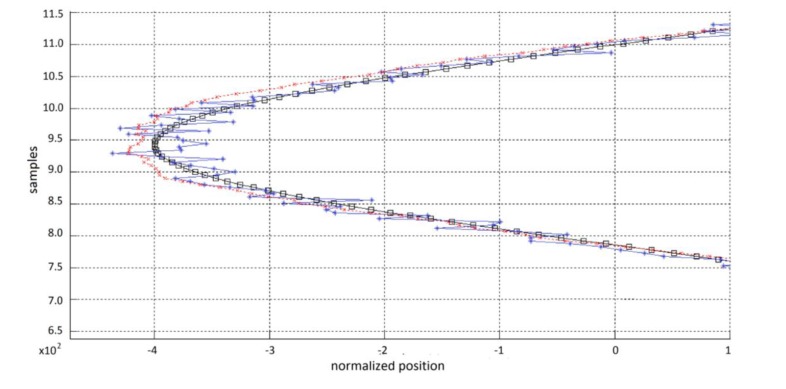
Kalman filtering as state estimator in mouse control and jitter removal; target movement (black line), observed movement (blue line), and filtered movement (red line).

Noise process variance was established from measurements using the data from gyroscope at rest, which is considered the process noise. Noise measurement variance was determined from data obtained during the experiment related to measurement or sensor noise. Figure 12 shows an example of plots obtained from a movement, with the filtered signal, unfiltered signal and desired trajectory, for comparison purposes.

### 3.3. Double Blinking Detection

**Figure 13.**
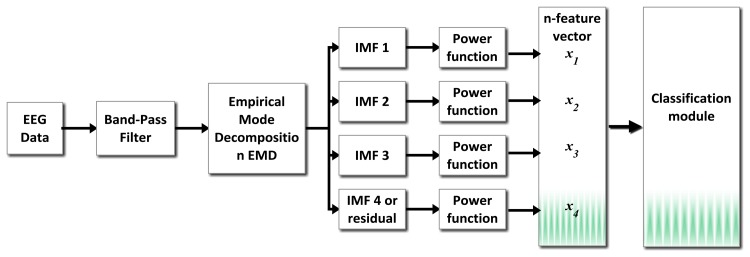
General scheme of detection system proposed.

**Figure 14.**
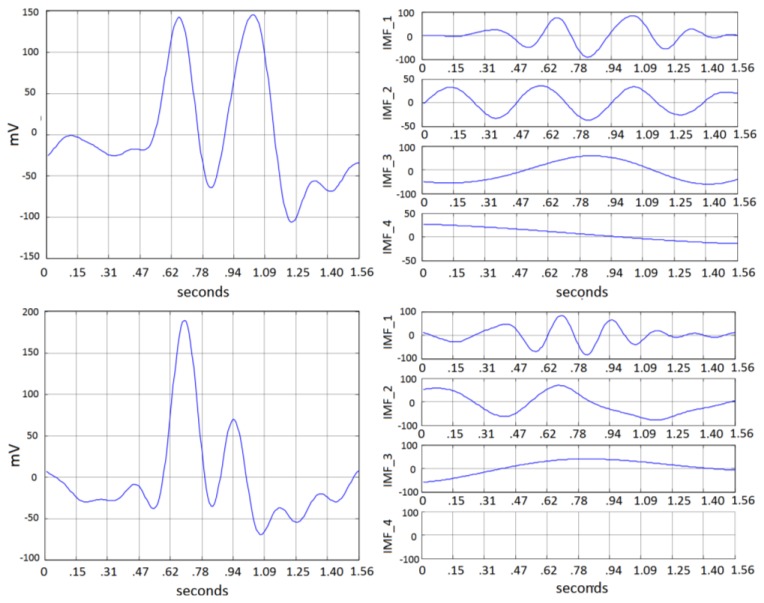
Typical EMD decompositions (**Right**) for blinking events (**Left**) from two different subjects under test.

Amplitude and time duration of double blinking vary among different person depending on physiological characteristics and intensity of the action. A test window of 2 s (256 samples) starts when a mark is sent to the recording system, which is then processed through the described EMD decomposition. A complete view of the system is depicted in Figure 13. The estimated data rate is about 20 bits/min, based on the considered window length plus 1 s for debouncing. Typical double blinking was found to be formed from 1 to 3 IMF and residual. Figure 14 shows typical decomposition for two blinking events from two different subjects. Features are obtained from the energy of each IMF and residual. The obtained feature vectors are then fed into a Mahalanobis-distance classifier. System test was performed using fold validation.

## 4. Experimental Setup and Results for Double Blinking Signal Detection and Gyroscope Data Processing

**Figure 15.**
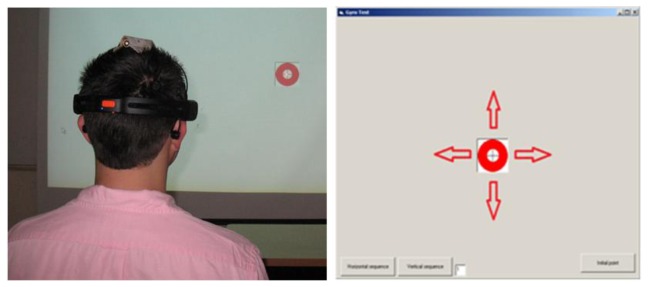
Experimental setup of EEG-based mouse emulation.

**Figure 16.**
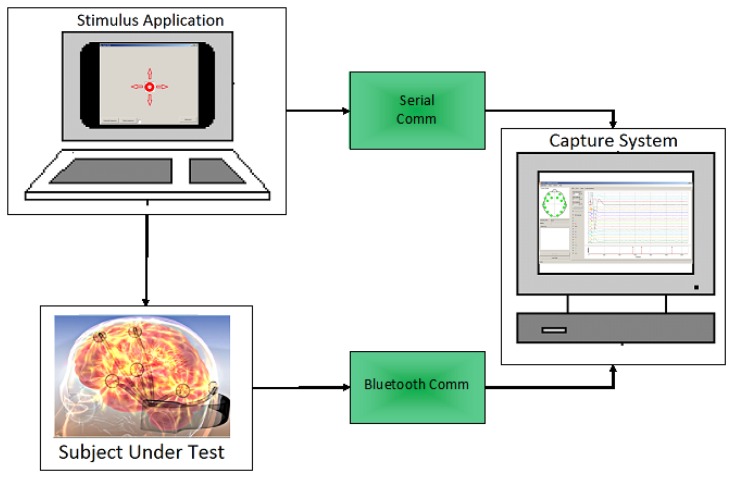
Testing setup system.

Subjects under testing were seated in a comfortable position using the Emotiv headset with a laser pointer attached at the top, as shown in Figure 15. A simple application developed in visual basic, showed a red circle moving through the screen following horizontal and vertical displacements, with a linearly increasing speed. The subject was instructed to follow as closely as possible the red circle with the pointer. EEG and gyroscope data were recorded simultaneously. Additionally, the subject was told to do a double blinking when a black circle appears in the screen. In that instant, the application sends a marker to the recording system. Thus, the test considers the worst case scenario in which the user is moving the head and doing a double blinking simultaneously. This case would rather occur in a practical situation, because the user usually stops the movement before doing a click with the mouse. Testing setup system is depicted in Figure 16.

**Figure 17.**
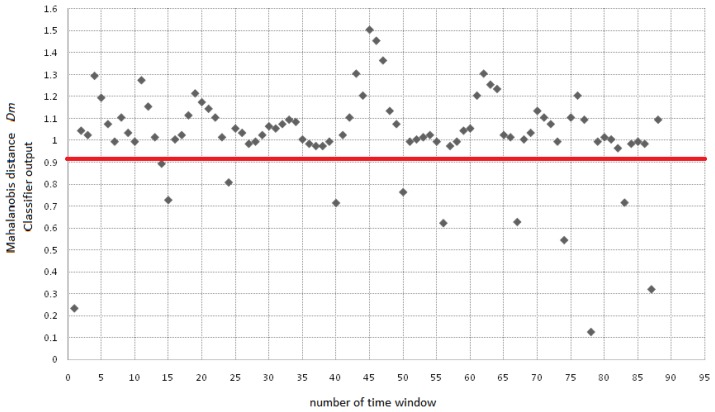
Range of double blink detection for the classification module.

System test was performed using fold validation, dividing in a random way the data set in two groups of 10 vectors each. The system is tested using 5 complete sequence of 91 s, in which 10 double blinking events occur randomly. A double blinking event was experimentally found to fall inside a distance value of 0.95. The Mahalanobis distance is defined by Equation (17), where ***x*** is the feature vector, *μ* is the mean vector and Σ is the covariance matrix. The example sequence in Figure 17 is processed using a sliding window of 256 samples, and then fed into the classification module. The sequence for this subject corresponds to 91 s, which have been analyzed through 90 windows of 256 samples sliding 128 samples. Red line indicates a decision threshold used to make the decision on whether or not a blinking was detected:

(17) $$D_{m}={\left(x-\mu \right)}^{T}\sum^{-1}(x-\mu )$$

**Figure 18.**
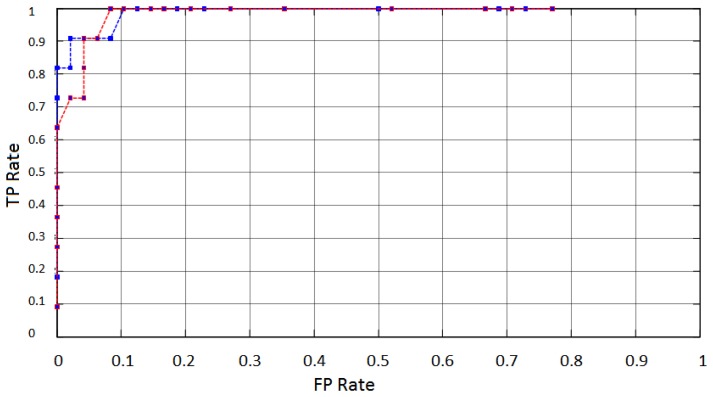
Average ROC curves obtained through measurements from AF4 (blue) and AF3 (red) electrodes.

System performance is analyzed through a Receiver Operation Characteristic plot (ROC) [68], which indicates the FPR (False Positive Rate) *versus* the TPR (True Positive Rate). Figure 18 shows the ROC average curves obtained through measurements from AF4 (blue) and AF3 (red) electrodes. The ROC curves were obtained by moving a threshold across the decision range from 0 to 1.91, in the Mahalanobis distances D_m_ obtained from the classifier output. The curves indicate a common tendency of an increasing rate of true positives events with simultaneous increasing rate of false positives. A small increase of the FP rate compared to the variation of TP rate can be noticed. Changes on FPR and TPR are results of variations in threshold value.

**Figure 19.**
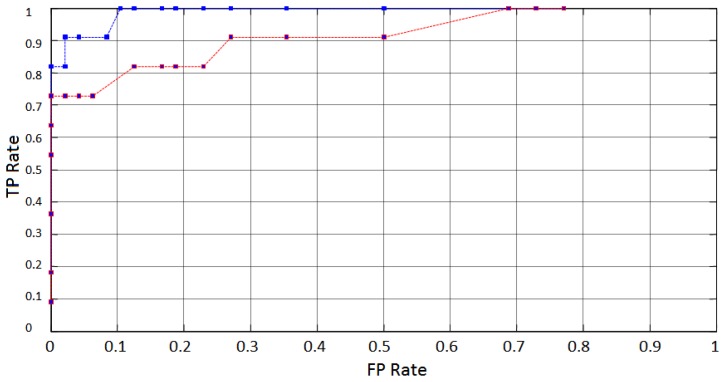
Average ROC curves obtained for EMD decomposition from AF4 (blue) and for Wavelet decomposition (red) from the same electrode.

According to Table 1, a popular feature extraction technique widely used in blinking detection is DWT. The experiment described in this work was duplicated for comparison purposes, using DWT as a second approach of feature extraction using spectral analysis. A 5 level Daubechies-2 wavelet decomposition applied to the same test windows under similar conditions was used to provide time-scale information. Wavelet decomposition typically concentrated energy related to blinking actions at levels 2 through 5 (∼1 Hz to 8 Hz). A feature vector constructed with energy values obtained from the obtained wavelet coefficients was used as input to the same classifier under similar conditions. Figure 19 shows the average ROC curves obtained from both, EMD and DWT approaches. It can be seen that EMD applied on the same data performed better than DWT.

The Emotiv system could detect velocities in the range from 0 to 600 degrees per second with a 12 bit resolution ADC, with velocity increments up to 0.14 degrees per second. This variation is mapped to an integer range from 0 to 4,000. A simple approach for jitter removal reported in similar systems is the use of a dead band with a previously established threshold. It is evident that the use of a dead band implies losing precision in cursor location and speed. A direct evaluation based on simple experiments indicates that it would be necessary at minimum a dead-band of 10 steps to eliminate shakiness at best scenario and almost 30 steps for fast movements. In that scenario, the system would lose information on a range of variations from 1.4 degrees per second up to 4.2 degrees per second. Using the Kalman estimator, which takes in account the noise variance, the loss would be only in the order of 0.7 degrees per second.

## 5. Conclusions

We have presented an initial prototype of a mouse control which takes advantage of gyroscope and EEG signals obtained from the commercial Emotiv headset. Emulation of mouse-clicks using double blinking is detected using Empirical Mode Decomposition. Analysis on detection rate indicated that EMD provided an efficient, effective and quick computational tool, adequate to non-stationary signals. Despite movements of the testing subject during double clink event, the performance system shows excellent results. About the task of transforming gyroscope data into mouse device movements, Kalman filter as a state estimator for mouse position control and jitter removal offers a better approach to increase mouse pointer resolution in comparison to consider a threshold-based dead band to eliminate noise and shakiness. The proposed noise reduction method based on information available from multiple electrodes is a preprocessing technique which can be adapted to different EEG systems, when noise caused by head or body movement is required to be removed. Double blinking detection using a feature extraction technique based on Empirical Mode Decomposition provided a detection rate of 94.9% in average using a Mahalanobis-distance based classifier. Additional experiments exploring the incorporation of classifiers such as Support Vector Machine and Neural Networks are currently in progress.

## Figures and Tables

**Table 1.** Comparison of several reported methods for blinking detection using electrodes as element sensor.

**Reference**	**Pre-Processing**	**Features**	**Classifier**	**Performance Metrics**
N. Kurian, *et al*. 2013 [33]	None	Amplitude values	Thresholding	Not specified
T. Wissel, *et al*. 2011 [34]	Bessel filtering	Wavelet Transform	1NN/LDA/Neural Networks	90%–94%
R. Barea, *et al*. 2011 [35]	None	Wavelet Transform	Neural Networks	92%
B. Paulchamy, *et al*. 2012 [36]	Not specified	Wavelet Transform	Adaptive Noise Cancellation	Based on SNR values
L.F. Araghi, 2010 [37]	None	Wavelet Transform	ADALINE (adaptive linear neuron)	Not specified
P. Kumar, *et al*. 2008 [38]	None	Wavelet Transform	Thresholding by statistical parameters	Not specified
P. SenthilKumar, *et al*. 2008 [39]	None	Wavelet Transform	ADALINE (adaptive linear neuron)	Supression ratio: 3–71 dB
W. Hsu, *et al*. 2012 [40]	Surface Laplacian	Wavelet Transform	Support Vector Machine	84% average
X. Yong, *et al*. 2009 [41]	None	Morphological Component Analysis	Creation of dictionary/template	Not specified
J. Lin, *et al*. 2012 [42]	Not specified	FFT	Simple Threshold	Results in average time consumed: 4.15–13.35 min
H. Shahabi, *et al*. 2012 [43]	None	Kalman Filter modeling	Simple Threshold	98% modeling fitting
M.K.I. Molla, *et al*. 2012 [44]	None	EMD	Thresholding by statistical parameters	Not specified
L. Ming-Ai, *et al*. 2011 [45]	None	EMD	Simple Threshold	RRMSE against ICA: 0.1143 and 01186
T. Jung, *et al*. 2000 [46]	None	Statistical parameters/ICA/E-ICA	Threshold filtering	Expert manual evaluation
S. Woltering, *et al*. 2013 [47]	None	Statistical parameters	Correlation	Correlation values for several electrodes
P. Balaiah, *et al*. 2012 [48]	Not specified	Statistical parameters	ADALINE (adaptive linear neuron)	SNR average 10.29
H. Nolan, *et al*. 2010 [49]	Filtering not specified	Statistical parameters/ICA	Thresholding by statistical parameters	Specificity > 90%
H. Cai, *et al*. 2011 [50]	Not specified	ICA based features	Thresholding by statistical parameters	Correlation values: 0.8457
